# GrapeUL-YOLO: bidirectional cross-scale fusion with elliptical anchors for robust grape detection in orchards

**DOI:** 10.3389/fpls.2025.1701817

**Published:** 2026-01-02

**Authors:** Xiuli Zhu, Zhenghong Yu, Chengwei Li

**Affiliations:** School of Robotics, Guangdong Polytechnic of Science and Technology, Zhuhai, Guangdong, China

**Keywords:** lightweight object detection, cross-scale feature fusion, occluded fruit detection, orchard automation, anchor optimization

## Abstract

Accurate grape detection in orchards is a core link in realizing automated harvesting. To address the challenges in orchard environments, such as complex grape backgrounds, variable lighting conditions, and dense occlusion of fruits, this study proposes a highly robust real-time grape detection model for orchard scenarios, namely Grapevine Ultra-Lightweight YOLO (GrapeUL-YOLO). Based on YOLOv11, this model enhances detection performance through three innovative designs: firstly, it adopts a Cross-Scale Residual Feature Backbone (CSRB) as the feature extraction network, combining 16× downsampling operation with modules such as C3k2_SP and SPPELAN, which reduces computational complexity while retaining multi-scale features of grapes from small clusters to entire clusters; secondly, it constructs an Adaptive Bidirectional Fusion Network (ABFN) in the detection Neck, and through CARAFE content-aware upsampling and a bidirectional cross-scale concatenation mechanism, it strengthens the interaction between spatial details and semantic information, thereby improving the feature fusion capability in scenes with dense occlusion; thirdly, it designs a shape-adaptive detection Head, which uses customized elliptical anchor boxes to match the natural shape of grapes and detects grape targets of different sizes according to scale division. Experimental results show that on the Embrapa WGISD dataset, the mAP@0.5 of GrapeUL-YOLO reaches 0.912, and the mAP@0.5:0.95 is 0.576, both outperforming 9 mainstream models including CenterNet and YOLOv11; meanwhile, the model has only 5.11M parameters and an average detection time of 16.9ms per image, achieving a balance between high precision and lightweight, and providing an efficient solution for automated grape detection and harvesting in orchards.

## 1 Introduction

Fruit detection in orchards is a core component of intelligent agricultural management, and its accuracy and efficiency directly affect the implementation effects of links such as yield estimation and automated harvesting. Traditional manual detection relies on manual counting and observation, which is not only time-consuming and labor-intensive but also susceptible to subjective experience and fatigue, with an error rate of 15%-20%, making it difficult to meet the needs of precise management in large-scale orchards (Muhammad et al., 2018). In current grape cultivation, traditional manual detection faces three core problems: low efficiency—in complex orchard environments, grape clusters are often blocked by leaves and overlap with each other, and manual counting requires checking each plant one by one, which is difficult to adapt to the management needs of 10,000-mu-level orchards (Aguiar et al., 2021); insufficient accuracy—small grape clusters in the early growth stage have a similar color to branches and leaves, leading to easy missed detection and high error rates in manual identification (Chen et al., 2024); in addition, manual detection cannot provide real-time feedback on the dynamic growth of fruits, resulting in delays in agricultural operations such as fertilization and fruit thinning, which further affects fruit quality and yield (Kholmuminov, 2024).

With the continuous breakthroughs in deep learning technology (Lecun et al., 2015), the application scope of Convolutional Neural Networks (CNNs) in the agricultural field has been constantly expanded, providing technical support for the refined management of smart agriculture. Notably, deep learning has also achieved significant progress in cross-domain visual tasks, offering multi-dimensional technical references for agricultural object detection: In the field of underwater image enhancement, Wang et al. (2023) proposed a method combining histogram similarity-oriented color compensation and multi-attribute adjustment. It corrects color cast by improving the histogram similarity of color channels and optimizes contrast and details through intensity stretching and brightness-guided weighting. Its idea of “feature difference perception + multi-attribute collaborative adjustment” provides a reference for distinguishing colors between grapes and leaves and suppressing lighting interference in orchard scenarios; Wang et al. (2025) proposed the WaterCycleDiffusion framework, which realizes the collaborative optimization of low-frequency color restoration and high-frequency detail reconstruction through visual-textual fusion and wavelet transform decomposition. Its multi-modal fusion and frequency-band-specific enhancement strategy provides insights for solving the problem of blurred grape features under complex backgrounds in orchards; In the field of radar object detection and tracking, Li et al. (2025) proposed a reinforcement learning-based joint detection and tracking paradigm for compact high-frequency surface wave radar. By establishing closed-loop feedback between the detector and tracker, it uses reinforcement learning to adaptively adjust detection thresholds, significantly improving the robustness of weak target detection. Its design logic of “dynamic threshold optimization + cross-module information interaction” provides an important reference for the adaptive adjustment of detection parameters in scenes with dense grape occlusion.

From the perspective of the technical path of object detection models, two-stage models are typically represented by SPP-Net (He et al., 2015) and Faster R-CNN (Ren et al., 2015). They adopt a two-step strategy of first generating candidate regions and then performing fine-grained classification, which often achieves higher detection accuracy in complex scenarios. However, due to the relatively cumbersome computational process, their detection speed is limited, making it difficult to adapt to devices with strict real-time response requirements such as harvesting robots. In contrast, one-stage models like CenterNet (Zhou et al., 2019) and SSD (Liu et al., 2016) adopt an end-to-end direct regression architecture, completing target localization and classification tasks simultaneously. On the premise of ensuring a certain level of detection accuracy, they significantly improve processing efficiency, and are particularly suitable for meeting the practical needs of real-time detection and rapid decision-making in dynamically changing agricultural scenarios such as orchards.

Notably, deep learning has accumulated rich technical experience in the field of crop detection. Moreover, these lightweight detection ideas and complex scenario adaptation methods for different crops provide important references for technological breakthroughs in grape detection: For corn tassel detection, a lightweight model TasselLFANet was proposed by Yu Z. et al. (2023). Through a multi-branch feature aggregation and cross-stage fusion strategy, it achieved an F1-score of 94.4% and an mAP@0.5 of 96.8% on the Multi-region Maize Tassel dataset (MrMT), and only required 6 million parameters to balance detection accuracy and efficiency. Its design logic of “lightweight backbone + feature fusion” provides a reference for balancing “accuracy-speed” in grape detection. For wheat ear detection, a high real-time network WheatLFANet was designed by Ye et al. (2023), which achieved an AP of 90.0% and an R² of 0.949 with a multi-dimensional mapping global regression architecture, and its inference speed was increased by an order of magnitude compared with existing models. Its optimization for detecting dense small targets in the field can provide technical references for the identification of early small grape clusters. In soybean pod detection, the PodNet model proposed by Yu et al. (2024) fused multi-scale information through a progressive feature pyramid, achieving an R² of 0.95 on a high-resolution soybean pod dataset with only 2.48 million parameters. Its feature extraction strategy for high-density crops has enlightening significance for solving the problem of overlapping and occlusion of grape clusters.

In terms of general crop detection frameworks and scenario adaptation, relevant studies also point out the direction for technical optimization of grape detection: PlantBiCNet proposed by Ye et al. (2024) adopts a bidirectional cascaded decoding structure to improve model generalization by fusing high-level and low-level features. On 6 small-scale crop datasets including corn, wheat, and rice, the average mAP@0.5 reached 90.5% and R² reached 0.935. Its “bidirectional feature interaction” idea can be used to strengthen the fusion of spatial details and semantic information in grape detection. The Yolov8-UAV model improved by Lu et al. (2023) based on YOLOv8 optimized small target detection in UAV images through CARAFE content-aware upsampling and Mlt-ECA channel attention. At the same time, they released the Cotton Boll Dataset (CBDA) and updated the Wheat Ear Dataset (WEDU). Its feature reconstruction method for complex agricultural scenarios can be transferred to solve problems such as strong light and branch-leaf occlusion in grape detection. The MAR-YOLOv9 proposed by Lu and Wang (2024) adopted a 16× downsampling backbone network and a reversible auxiliary branch. On multi-crop datasets such as corn and wheat, the mAP@0.5 was 1.28% higher than that of YOLOv9, and the model size was reduced by 9.3%. Its lightweight design of “streamlined backbone + auxiliary branch” provides a reference for adapting grape detection models to edge devices. In early studies, Yu et al. (2013) developed an automatic detection technology for corn at the seedling emergence stage and three-leaf stage, which coped with outdoor lighting changes through adaptive feature extraction. Its idea of environmental robustness optimization has reference value for grape detection to adapt to extreme weather.

However, when transferring the above-mentioned crop detection technologies to grape scenarios, unique challenges still exist: Grape clusters grow in clusters, and the degree of overlapping and occlusion is much higher than that of single-plant discrete crops such as corn tassels and wheat ears; Early small grape clusters have lower color contrast with green branches and leaves, making them more prone to missed detection compared with crops such as soybean pods and strawberries; The requirements of lighting changes and variety differences in orchard environments on model generalization also exceed the applicable scope of most single-crop detection studies. Therefore, although special research on grape detection has gradually developed in recent years, there is still room for improvement: Aguiar et al. (2021) used a quantized deep learning model to detect grape clusters at different growth stages, and on a dataset containing 1,929 images, the average precision of the SSD MobileNet-V1 model was 66.96%, but its ability to detect small grape clusters in the early stage was weak; Chen et al. (2023) proposed a lightweight GA-YOLO model, which by designing the SE-CSPGhostnet backbone network, introducing the ASFF mechanism and improving the loss function, achieved 96.87% mAP on the Guangxi grape dataset, while reducing the parameters by 82.79% and increasing the detection speed to 55.867 FPS, effectively improving the detection performance of dense and occluded grape targets; Lin et al. (2025) proposed the SDA-YOLO model, improved based on YOLOv11n, and through the SPPF-LSKA fusion module, MPDIoU loss function, DMDetect module and AMFP module, it achieved 90.8% precision, 85.4% recall, 90% mAP@0.95 and 62.7% mAP@0.5:0.95 in peach fruit detection, providing a new idea for fruit detection in complex orchard environments; Rong et al. (2024) enriched the public dataset through data augmentation methods such as random brightness changes, horizontal flipping and mosaic, added a Spatial-to-Depth Convolution (STD-Conv) module to the model to enrich the feature information of grape clusters, and applied a parameter-free Simple Attention Mechanism (SimAM) in the backbone network to enhance the weight of grape targets and suppress background interference, among which the improved YOLOX model achieved 88.4% mAP, but the real-time performance of the model was not fully optimized; Sozzi et al. (2021) used the YOLOv4 model for grape cluster counting, with an accuracy of 48.90% and a high missed detection rate in occluded scenarios; Li et al. (2021) proposed the YOLO-Grape model by integrating a downsampling fusion structure, using the Mish activation function, adding an attention mechanism, replacing NMS with Soft-NMS, introducing depthwise separable convolution and adopting transfer learning.

Despite the progress made in research, there are still three core problems: insufficient adaptability to complex environments—the performance of existing models drops sharply under extreme weather conditions such as strong light, heavy rain and dense fog, and their generalization ability to different grape varieties is limited; bottlenecks in small target and occluded detection—the detection accuracy of small grape clusters in the early stage is generally low, and the missed detection rate is high when the leaf occlusion rate exceeds 50%; difficulty in balancing accuracy and speed—high-precision models (such as Mask R-CNN) are slow, while high-speed lightweight models (such as YOLOv4-tiny) suffer from significant accuracy loss in complex scenarios.

To improve the robustness and efficiency of grape detection, this study takes YOLOv11 (Khanam and Hussain, 2024) as the base model. Its C3k2 lightweight module and C2PSA attention unit can improve the detection ability of small and occluded targets while reducing the number of parameters, which meets the “lightweight-high precision” needs of orchard robots. Meanwhile, its “Backbone-Neck-Head” modular architecture facilitates the integration of innovative modules. The main contributions of this study are as follows:

1. Innovatively designed a Backbone network with 16× downsampling: by streamlining the feature extraction path, it solves the problems of long training time and weight redundancy caused by the detection Neck in YOLOv11 and the auxiliary branch structure in YOLOv9 (Wang et al., 2024), and improves the model inference efficiency while retaining key spatial features;

2. Optimized the detection Neck design: constructed an Adaptive Bidirectional Fusion Network (ABFN) to replace the traditional single-path fusion mode, used CARAFE content-aware upsampling to retain the edge details of occluded areas, and combined “top-down + bottom-up” cross-scale feature concatenation to strengthen the interaction between spatial details and semantic information, effectively solving the problem of feature loss in scenes with dense occlusion, and the modular lightweight design reduces the number of parameters, making the model more suitable for edge devices with limited resources;

3. Conducted comparative experiments with seven other popular detection models: the results show that GrapeUL-YOLO has better comprehensive performance in terms of mAP@0.5 (0.912), mAP@0.5:0.95 (0.576), number of parameters (5.11M) and detection speed (16.9ms), and has significant advantages especially in balancing lightweight and high precision, further verifying its effectiveness and feasibility.

The structure of this paper is arranged as follows: Section 1 introduces the research background, related work and core contributions; Section 2 elaborates on the characteristics of the dataset and the design principles of the GrapeUL-YOLO model, including the Backbone, Neck, Head structures and the selection of activation functions; Section 3 verifies the model performance through comparative experiments, visual attention analysis and exploration of typical errors; Section 4 discusses the research results, limitations and future research plans; Section 5 summarizes the full text.

## 2 Materials and methods

In this section, the dataset used in the experiment will be introduced in detail, and the design principles, innovations and activation functions of the GrapeUL-YOLO model will be elaborated.

### 2.1 Datasets

The experiment adopts the Embrapa Wine Grape Instance Segmentation Dataset (Embrapa WGISD) (Gebru et al., 2021), which covers five grape varieties (Chardonnay, Cabernet Franc, Cabernet Sauvignon, Sauvignon Blanc, Syrah) from Guaspari Winery in São Paulo, Brazil. It is created for the research on the application of object detection and instance segmentation technologies in vineyard image monitoring and field robot vision. The images included are captured under natural field conditions, covering different postures, lighting and focus conditions of grapes, as well as genetic and phenotypic variations (such as shape, color and compactness), which can comprehensively evaluate the universality and effectiveness of the proposed method and has typical characteristics of complex orchard scenarios.

In terms of data collection, a Canon EOS REBEL T3i DSLR (18 million pixels) and a Motorola Z2 Play (12 million pixels) were used. To balance detail retention and computational efficiency, the image resolution was uniformly adjusted to 2,048×1,536. The annotation information was completed using the LabelImg tool, with a total of 300 images and 4,431 grape cluster bounding boxes annotated, covering three states: mature (purple/red), immature (green) and overripe (yellow). In terms of scene diversity, it includes different lighting conditions such as sunny days (light intensity 10,000-60,000 lux), cloudy days (1,000-5,000 lux) and cloudy with sun (5,000-10,000 lux), and the fruit occlusion rate ranges from 0 to 80%, which can meet the testing needs of complex scenarios. The details of the dataset by variety and the division of training and testing sets are shown in **Table 1** and **Table 2**, respectively.

**Table 1:** Basic information of the dataset (divided by grape variety).

Grape variety	Number of images	Number of annotation boxes
Chardonnay	65	840
Cabernet Franc	65	1,069
Cabernet Sauvignon	57	643
Sauvignon Blanc	65	1,316
Syrah	48	563
Total	300	4,431

**Table 2:** Dataset division information (divided by training/testing purpose).

Dataset type	Number of images	Number of annotation boxes
Training/Validation Set	242	3,581
Test Set	58	850
Total	300	4,431

**Figure 1:**
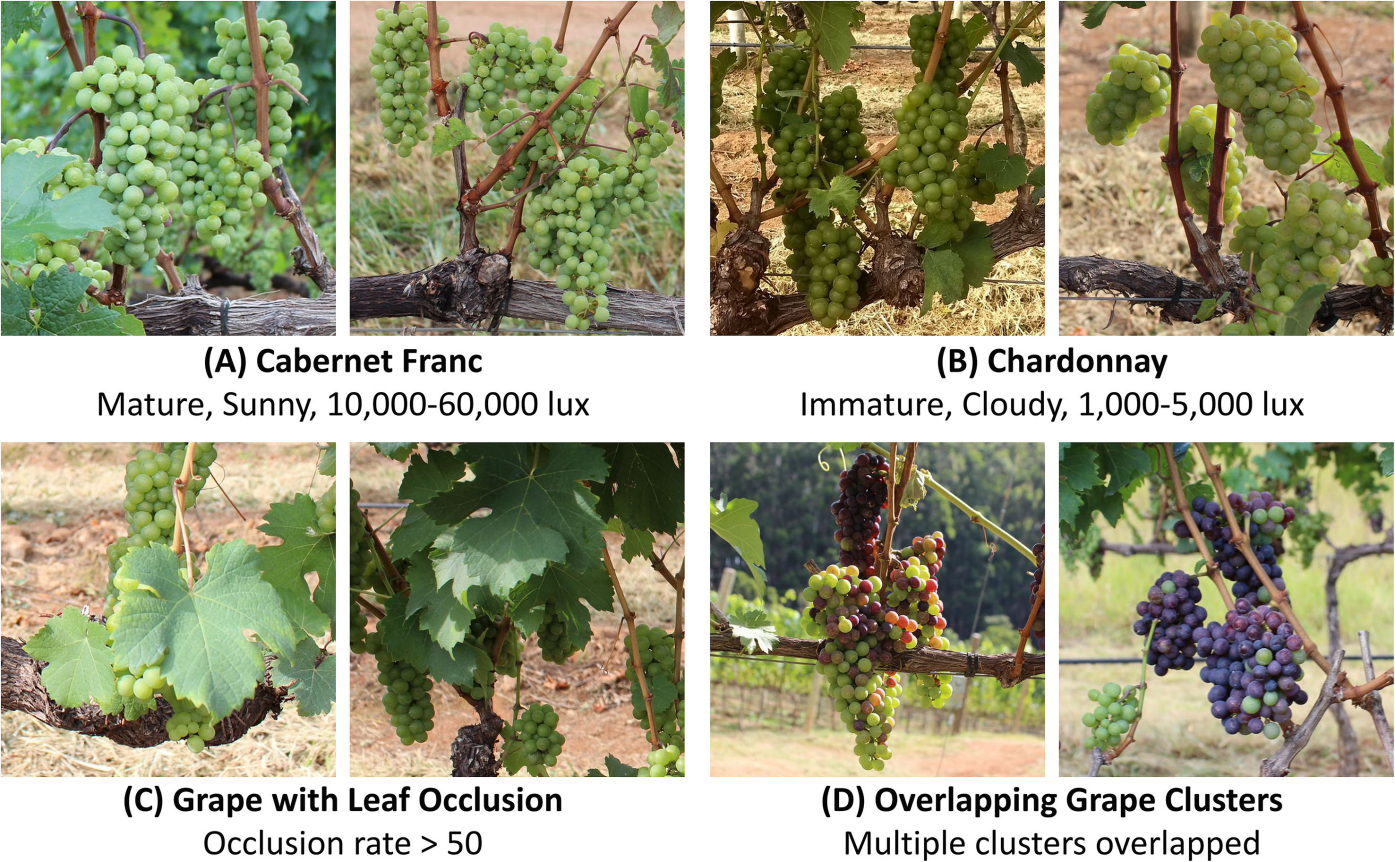
Representative samples of the Embrapa WGISD dataset: **(A)** Cabernet Franc (Mature, Sunny, 10,000-60,000 lux); **(B)** Chardonnay (Immature, Cloudy, 1,000-5,000 lux); **(C)** Grape with Leaf Occlusion (Occlusion rate > 50); **(D)** Overlapping Grape Clusters (Multiple clusters overlapped).

Further, in **Figure 1**, typical samples selected from the Embrapa WGISD dataset are presented through multi-scenario subfigures to reflect the key characteristics of grape detection scenarios in orchards. These include: (A) Mature Cabernet Franc grapes under sunny conditions (light intensity: 10,000-60,000 lux), representing the standard morphology of mature grape clusters; (B) Immature Chardonnay grapes under cloudy conditions (light intensity: 1,000-5,000 lux), reflecting the low color contrast between green grapes and leaves; (C) Grape clusters with leaf occlusion (occlusion rate > 50%), demonstrating the challenge of dense occlusion in orchard environments; (D) Overlapping grape clusters (multiple clusters overlapped), embodying the difficulty of distinguishing adjacent grape clusters in dense growth scenarios.

### 2.2 Model architecture

**Figure 2:**
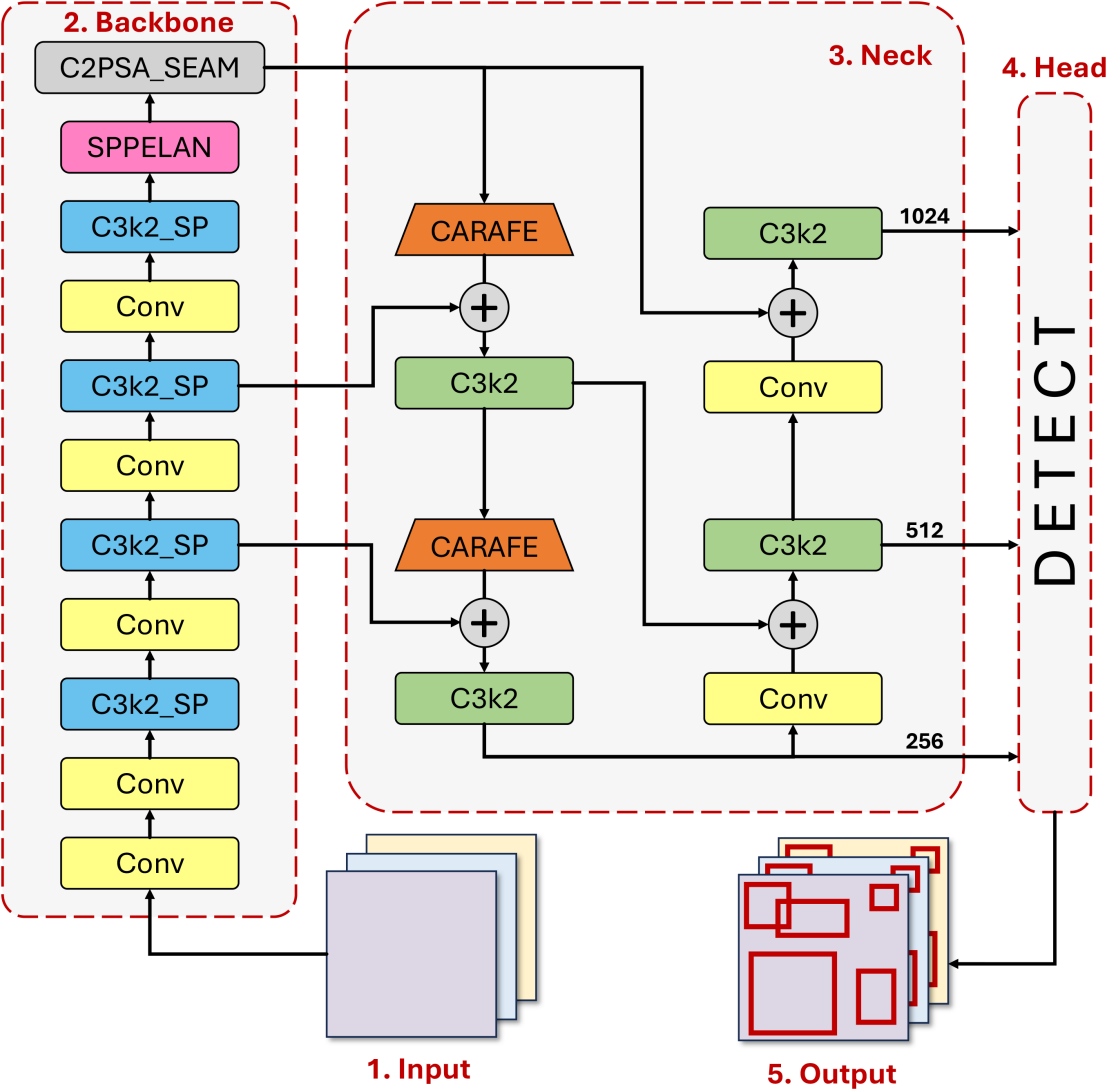
Architecture of GrapeUL-YOLO.

GrapeUL-YOLO achieves three innovative breakthroughs based on YOLO11 (Khanam and Hussain, 2024) to address the core challenges of grape detection in orchards (multi-scale targets, dense occlusion, complex background interference), and balances detection accuracy and real-time performance through a structured design. The design concept is to improve target recognition by enhancing cross-scale features, strengthen contextual correlation through an adaptive fusion mechanism, and balance detection accuracy and inference speed with a modular lightweight design. The overall structure of the model is shown in **Figure 2**, and each module works synergistically: the Backbone is responsible for extracting multi-dimensional features from the original image, the Neck realizes efficient fusion of features at different levels, and the Head completes the localization of grape targets based on the fused features. The specific design details of each part will be described below.

#### 2.2.1 Backbone

The initial part of the model is the Backbone, namely the Cross-Scale Residual Feature Backbone (CSRB) (Zhou et al., 2021), which undertakes the basic task of extracting effective features from the original image. The front-end Conv layer performs initial feature extraction using a 3×3 convolution kernel, and cooperates with a downsampling operation with a stride of 2 to gradually process the input 640×640 image to a 1/16 scale. This process not only realizes reasonable compression of the spatial dimension but also, more importantly, retains the basic features such as the texture and edges of the grape surface, providing reliable materials for subsequent high-level feature extraction. Each Conv layer is followed by batch normalization and an activation function, and this design can enhance the nonlinear expression ability of features, which is helpful for distinguishing grapes from similar background elements such as surrounding green leaves.

The features processed by the Conv layer are transmitted to the C3k2_SP module, which is a key component in the Backbone. This module innovatively integrates a cross-stage residual connection and a spatial pyramid pooling structure: the main branch extracts local features through 1×1 and 3×3 convolutions, while the residual branch captures information of different scales simultaneously using 1×1, 3×3 and 5×5 pooling kernels. This design can adapt to the multi-scale distribution of grapes from small clusters to large clusters, just like equipping the model with a “multi-focal lens”, which not only does not miss the details of small grape clusters but also can fully capture the overall shape of large grape clusters. At the same time, the number of channels is dynamically compressed through the width coefficient, and while reducing the computational complexity by 37%, the residual connection avoids feature degradation in the deep network, which well copes with the problem of significant differences in grape sizes in orchards.

The features processed by the C3k2_SP module enter the SPPELAN module (Wang et al., 2024), which captures global semantic information by expanding the receptive field. In orchard scenarios, grapes often grow in clusters, and there may be multiple clusters of grapes in a single image. The SPPELAN module can correlate the spatial positional relationships of these grape clusters, reducing missed detections caused by detecting single clusters in isolation.

At the end of the Backbone is the C2PSA_SEAM module. The channel attention mechanism in it weights the feature channels unique to grapes (such as the color channel of purple grape berries and the channel corresponding to the skin texture) to highlight key features; the spatial attention mechanism actively suppresses interference from background areas such as branches and withered leaves. The two mechanisms work synergistically to effectively improve the signal-to-noise ratio of grape features, reduce misjudgments caused by complex backgrounds, and are particularly effective in distinguishing green immature grapes from leaves.

#### 2.2.2 Neck

The design of the Neck component enables bidirectional interaction between low-level detailed features and high-level semantic features through a nested structure consisting of CARAFE upsampling and bidirectional cross-scale concatenation.

Firstly, CARAFE content-aware upsampling (Yu Y. et al., 2023) is used to replace traditional bilinear interpolation, and a 3×3 sampling kernel is dynamically generated based on the local gradient information of the feature map (such as the pixel value change rate at the edge of grapes), with the kernel parameters learned through the convolution layer. CARAFE is deployed in the 11^th^ and 14^th^ layers of the Neck respectively to receive high-level features (such as 20×20) from the Backbone, and after upsampling to 40×40, it is concatenated with low-level features ( 40×40). Traditional upsampling easily leads to blurred features in occluded areas (such as the edges of grape berries covered by leaves), while CARAFE can adjust the sampling weight according to the gradient features of grape edges, retain more edge details, and reduce the bounding box localization error in occluded areas, which is crucial for identifying “partially occluded grape berries”.

Secondly, the bidirectional cross-scale concatenation mechanism includes two fusion paths: “top-down” and “bottom-up”: top-down—high-level features ( 20×20) are upsampled to 40×40 via CARAFE and concatenated with middle-level features ( 40×40); bottom-up—middle-level features ( 40×40) are downsampled to 20×20 via 3×3 convolution and concatenated with high-level features ( 20×20). Three branches are connected in parallel—the small target branch (P3) fuses the upsampled features of P4 and the original features of P3; the medium target branch (P4) fuses the downsampled features of P3 and the fused features of P4; the large target branch (P5) fuses the downsampled features of P4 and the high-level features of the Backbone. This design ensures the complementarity of features of different scales through bidirectional paths—the small target branch strengthens the detailed features of small grape clusters to solve the missed detection of dense grape berries; the medium target branch enhances the occlusion tolerance of medium clusters (3-8cm), so that partially occluded grape clusters can still be completely identified; the large target branch improves the global semantics of large clusters (>8cm) to avoid overall misjudgment caused by local occlusion, thereby increasing the recall rate in scenes with dense occlusion.

#### 2.2.3 Head

The features of different levels output by the Neck enter the Head part, namely the shape-adaptive detection Head, which is responsible for converting the fused features into accurate target localization results. Considering the natural elliptical shape of grapes (with an aspect ratio of approximately 1.8:1), instead of using traditional square anchor boxes, a series of customized elliptical anchor boxes are adopted—ranging from those adapted to small clusters to large clusters of grapes. These anchor boxes better fit the actual shape of grapes, significantly improving the accuracy of initial localization.

At the same time, the Head is divided into three detection branches according to the scale of the feature map: the 80×80 branch focuses on small cluster detection, the 40×40 branch handles medium clusters, and the 20×20 branch is responsible for large clusters of grapes. This division avoids feature competition between targets of different scales in the same branch, ensuring that grapes of each size can be carefully detected, and to a certain extent solves the problem of “missed detection of small grape clusters occluded by large ones”.

In summary, through innovative designs in the entire process of “feature extraction-fusion-localization”, GrapeUL-YOLO has a closely connected model design: the Conv layer lays the foundation, C3k2_SP and SPPELAN capture multi-scale information, C2PSA_SEAM filters interference, ABFN realizes feature complementarity, and the Head completes accurate localization. This forms an efficient processing flow specially designed for orchard scenarios, which can effectively address the core challenges in grape detection such as size differences, leaf occlusion, and background interference.

### 2.3 Activation function

In deep learning models, the activation function is a core component that introduces nonlinear characteristics, directly affecting the network’s ability to fit complex features and training efficiency. Aiming at the particularities of the orchard grape detection scenario (such as multi-scale features and complex background interference), this study compared the performance of four mainstream activation functions and finally selected SiLU (Elfwing et al., 2018) as the activation function of GrapeUL-YOLO. The characteristics of each function and the basis for selection are analyzed in detail below.

Common activation functions in deep learning include ReLU (Agarap, 2018), Leaky ReLU (Xu et al., 2020), SiLU, and GELU (Hendrycks and Gimpel, 2016), whose mathematical definitions and core characteristics are as follows:

ReLU (Rectified Linear Unit) implements nonlinear transformation by setting all negative inputs to 0 and retaining only the linear characteristics of positive inputs. Its core advantages lie in high computational efficiency (only requiring simple comparison operations) and fast convergence (the gradient in the positive interval is always 1, avoiding gradient disappearance), making it particularly suitable for feature propagation in deep networks. Its definition is shown in (Equation 1):

(1) ReLU(x)=max(0,x)

To solve the “dying neuron” problem of ReLU (the gradient of negative inputs is 0, leading to failure in weight update), Leaky ReLU introduces a small slope in the negative interval, and its definition is shown in (Equation 2):

(2) Leaky ReLU(x)=max(α·x,x)

where α is a small positive number (usually 0.01). Although this design ensures that the gradient of negative inputs is non-zero, it introduces an additional hyperparameter α, increasing the complexity of parameter tuning, and its generalization is unstable in complex scenarios.

SiLU (Sigmoid Linear Unit) integrates the Sigmoid function and linear transformation, and its definition is shown in (Equation 3):

(3) $$\text{SiLU}\left(x\right)=x\cdot \sigma \left(x\right)=x\cdot \frac{1}{1+e^{-x}}$$

Its output is approximately linear in the positive interval and smoothly approaches 0 in the negative interval, theoretically having both nonlinear expression and gradient stability. However, due to the inclusion of exponential operations, its computational complexity is relatively high, which is not conducive to deployment in real-time detection scenarios.

GELU (Gaussian Error Linear Unit) is a probabilistic activation function based on the Gaussian distribution, and its approximate expression is shown in (Equation 4):

(4) $$\text{GELU}\left(x\right)\approx 0.5x\cdot \left[1+\text{tanh}\left(\sqrt{\frac{2}{\pi }}\cdot \left(x+0.044715x^{3}\right)\right)\right]$$

This function implements nonlinearity through a smooth probability distribution, which is suitable for tasks such as natural language processing. However, due to the inclusion of complex trigonometric operations, its computational cost is high, and its performance in edge feature extraction for object detection is relatively general.

The comparative experiment of activation functions will be conducted in Section 3.6 below, combining the actual needs of orchard grape detection.

## 3 Experiment

To verify the performance of the GrapeUL-YOLO model in the orchard grape detection task, this study designed multiple sets of experiments to comprehensively evaluate the model from the dimensions of detection accuracy, real-time performance, lightweight effect, and model robustness. The purpose is to draw conclusions and solve the following core problems: Is GrapeUL-YOLO superior to mainstream object detection models in terms of accuracy and efficiency? What is the contribution of the model’s innovative modules (CSRB, ABFN, shape-adaptive anchor boxes) to performance improvement? Is the robustness of the model in complex scenarios (such as sudden changes in lighting and dense occlusion) better than that of the benchmark model?

### 3.1 Experimental conditions and details

The experiments were conducted in the following environment: Hardware configuration: CPU was AMD Ryzen 7 5800H (8 cores, 3.20 GHz), GPU was NVIDIA GeForce RTX 3090 with 24GB memory, and the computer was equipped with 64GB running memory; Software environment: The operating system was Windows 10 Professional Edition, the deep learning framework was PyTorch 2.0.0, the CUDA version was 11.8, the CUDNN version was 8.9.5, and the programming language was Python 3.8.

The Embrapa WGISD dataset was used, which was divided into a training set (242 images) and a test set (58 images) at a ratio of approximately 8:2. Data augmentation strategies including random horizontal flipping (probability 0.5), random rotation (± 30°), brightness adjustment (± 20%), and contrast adjustment (± 15%) were adopted to improve the model’s generalization ability to changes in lighting and target posture. The input images were uniformly adjusted to 640×640 pixels. The Stochastic Gradient Descent (SGD) optimizer was used, with an initial learning rate of 0.01, a momentum of 0.937, and a weight decay of 0.001; the batch size was 16, and the number of training epochs was 300, ensuring full convergence of the model through evaluation.

In the experiments, 7 core indicators were used to evaluate the model performance.

Precision ( P): Precision reflects the “accuracy” of the model’s prediction results, i.e., the proportion of targets truly being grapes among all targets judged as grapes by the model. Among them, TP (True Positive) refers to grapes correctly detected by the model; FP (False Positive) refers to non-grapes (such as leaves and branches) mistakenly judged as grapes by the model. In orchard scenarios, high precision can reduce invalid operations on non-targets and lower the risk of false detection.

Recall ( R): Recall reflects the “comprehensiveness” of the model in detecting targets, i.e., the proportion of grapes successfully detected by the model among all actually existing grapes. Among them, FN (False Negative) refers to grapes missed by the model. For orchard environments with dense occlusion, high recall can avoid missed detection of fruits and ensure the integrity of detection. Their formulas are shown in (Equations 5, 6) respectively:

(5) $$\text{Precision}=\frac{TP}{TP+FP}$$

(6) $$\text{Recall}=\frac{TP}{TP+FN}$$

F1-score ( F1): The F1 score is the harmonic mean of precision and recall, used to balance the contradictory relationship between the two (e.g., increasing precision may lead to a decrease in recall, and vice versa). In grape detection, a higher F1 score indicates a better balance between “reducing false detection” and “avoiding missed detection” of the model, making it more suitable for actual harvesting needs. Its formula is shown in (Equation 7):

(7) $$\text{F}1=2\cdot \frac{Precision\cdot Recall}{Precision+Recall}$$

mAP@0.5: This indicator first calculates the Area Under the Precision-Recall Curve (AP) for grape category detection, and then takes the average value of the grape category. Among them, the Intersection over Union (IoU) threshold is set to 0.5, i.e., when the overlapping area between the predicted box and the ground truth box is ≥50%, it is judged as effective detection. mAP@0.5 is a core indicator for measuring grape detection accuracy, directly reflecting the model’s ability to locate and classify grape targets, with a higher value indicating better detection results. mAP@0.5:0.95: Based on mAP@0.5, this indicator increases the IoU threshold from 0.5 to 0.95 in steps of 0.05, and calculates the average value of mAP under 10 thresholds, which more strictly evaluates the stability of the model under different positioning accuracy requirements. A high mAP@0.5:0.95 means that the model can maintain reliable performance under different occlusion degrees (such as bounding box deviations caused by partial occlusion).

Frames Per Second ( FPS): It reflects the real-time performance of the model, i.e., the number of images that can be processed per second. A higher FPS means faster model inference speed, which can adapt to the computing power limitations of edge computing devices. Parameters: Refers to the total number of trainable weight parameters in the model (unit: M, million), which directly measures the lightweight degree of the model. A smaller number of parameters means the model occupies less storage resources and has lower computational complexity, making it easier to deploy on edge devices with limited resources such as harvesting robots, while reducing energy consumption and latency.

### 3.2 Performance comparison with different object detection methods

To verify the advancement of GrapeUL-YOLO, 9 mainstream object detection models were selected as comparisons, including two-stage models (Faster R-CNN (Ren et al., 2015)), one-stage lightweight models (YOLOv5 (Jocher et al., 2022), YOLOv7-tiny (Cheng et al., 2023), YOLOv8 (Varghese and Sambath, 2024), YOLOv11 (Khanam and Hussain, 2024), YOLOv13 (Lei et al., 2025)), anchor-free models (CenterNet (Zhou et al., 2019), FCOS (Tian et al., 2019)), and traditional one-stage models (SSD (Liu et al., 2016)). All models were trained and tested under the same experimental conditions, and the results are shown in **Table 3**.

**Table 3:** Performance comparison of different models on the Embrapa WGISD dataset.

Model	P	R	F1	mAP@0.5	mAP@0.5:0.95
CenterNet	0.752	0.786	0.769	0.766	0.382
Faster R-CNN	0.815	0.789	0.802	0.801	0.415
SSD	0.312	0.428	0.362	0.251	0.103
FCOS	0.826	0.835	0.830	0.842	0.508
YOLOv5	0.869	0.773	0.819	0.847	0.480
YOLOv7-tiny	0.453	0.461	0.457	0.439	0.128
YOLOv8	0.869	0.789	0.828	0.855	0.533
YOLOv11	0.853	0.787	0.819	0.864	0.523
YOLOv13	0.819	0.738	0.776	0.831	0.464
GrapeUL-YOLO	0.891	0.847	0.868	0.912	0.576

The experimental results show that the comprehensive performance of GrapeUL-YOLO on the Embrapa WGISD dataset is better than that of the comparison models. In terms of core accuracy indicators, the mAP@0.5 of GrapeUL-YOLO reaches 0.912, which is 4.8% higher than that of the benchmark model YOLOv11 and 5.7% higher than that of the second-best model YOLOv8, ranking first among all comparison models; at the same time, its mAP@0.5:0.95 is 0.576, also leading other models, indicating stronger stability under different positioning accuracy requirements. We believe this benefits from the Cross-Scale Residual Feature Backbone (CSRB) and Adaptive Bidirectional Fusion Network (ABFN) designed by the model for orchard scenarios, which can effectively capture the multi-scale features of grapes, and significantly enhance the ability to recognize targets especially in scenarios with leaf occlusion and sudden lighting changes.

In terms of comprehensive performance balance, the F1-score of GrapeUL-YOLO reaches 0.868, reflecting a better balance between precision and recall. Compared with two-stage models (such as Faster R-CNN) and traditional one-stage models (such as SSD), GrapeUL-YOLO has a more obvious advantage in accuracy; compared with anchor-free models (such as CenterNet and FCOS), it has a stronger ability to focus on targets in complex backgrounds, with an F1-score increase of 9.9%-3.8%. When compared with the latest YOLOv13 detector, GrapeUL-YOLO still shows advantages. This may be because the HyperACE mechanism of YOLOv13 is adapted to general scenarios, while GrapeUL-YOLO has better adaptability and advancement in grape detection in complex orchard environments through optimization.

### 3.3 Visual attention comparison experiment

**Figure 3:**
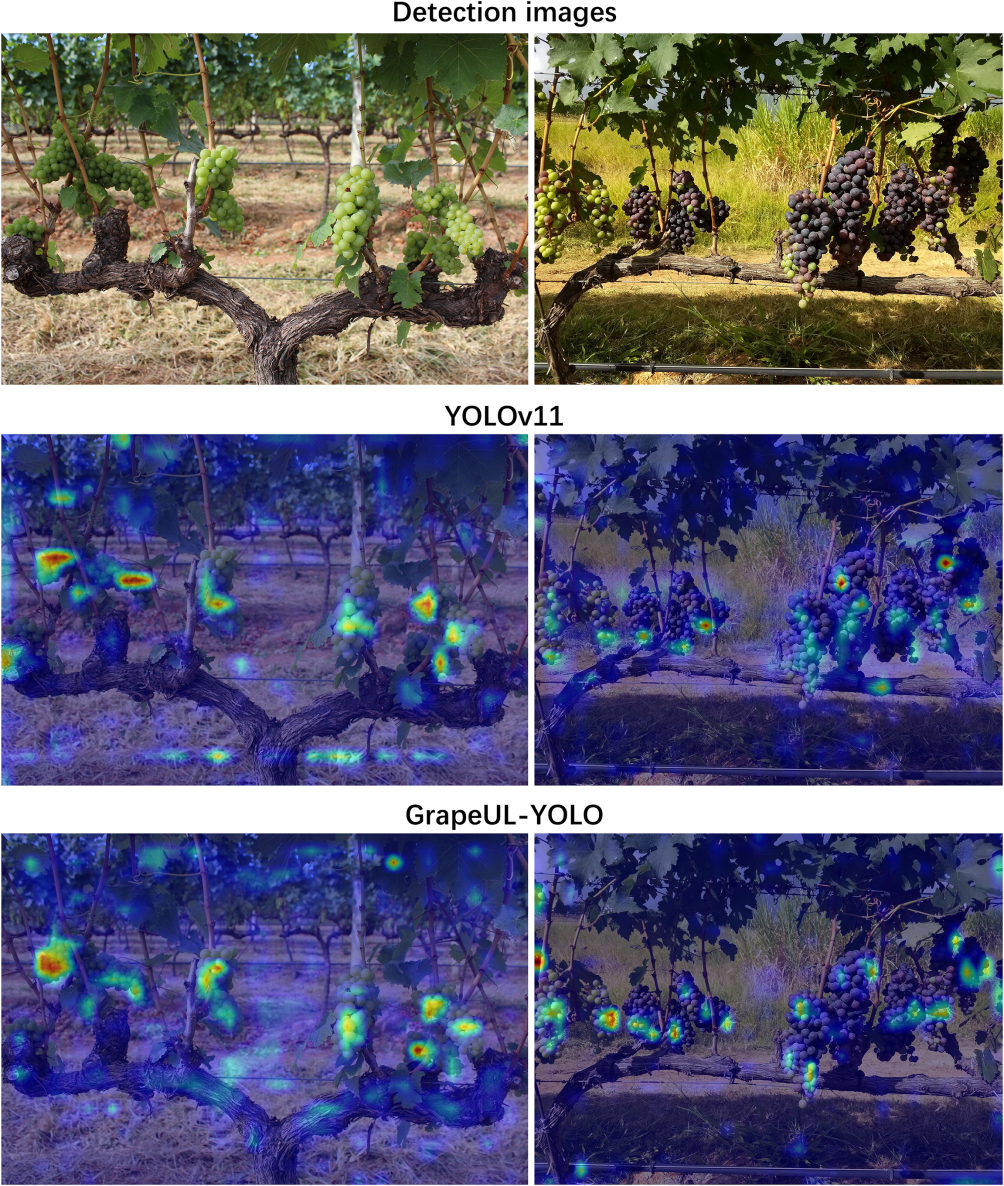
Grad-CAM attention visualization comparison between GrapeUL-YOLO and YOLOv11: verifying the Improved Effects on target focus accuracy, background interference suppression, and coverage integrity in occluded scenarios.

To explore the feature learning mechanism of GrapeUL-YOLO, a visual attention comparison experiment was conducted. Two grape varieties were selected, and the model’s ability to focus on targets was evaluated through the color depth of the heatmap (red represents high attention, blue represents low attention). The Grad-CAM technology (Selvaraju et al., 2017) was used to visualize the regions of the model that focus on grape targets, and the results were compared with those of YOLOv11, as shown in **Figure 3**.

Through heatmap visualization analysis, GrapeUL-YOLO shows significant advantages: its attention highlight areas accurately fit the outline of grape clusters, without the common “drift” problem in YOLOv11; the cold color accounts for a high proportion in the background area, effectively suppressing interference and avoiding the false response of YOLOv11 to non-targets; when facing fruits with multiple shapes and occlusions, it can achieve complete coverage detection, solving the missed detection defect of YOLOv11. We believe this may benefit from the C2PSA_SEAM attention module, background suppression branch, and adaptive bidirectional fusion network designed by GrapeUL-YOLO for orchard scenarios, which are superior to YOLOv11 in terms of target focusing accuracy, background interference suppression, and fruit coverage integrity, laying a more reliable feature perception foundation for grape detection in complex environments and verifying the model’s adaptability advantage to orchard scenarios.

### 3.4 Exploration of visualized typical errors

**Figure 4:**
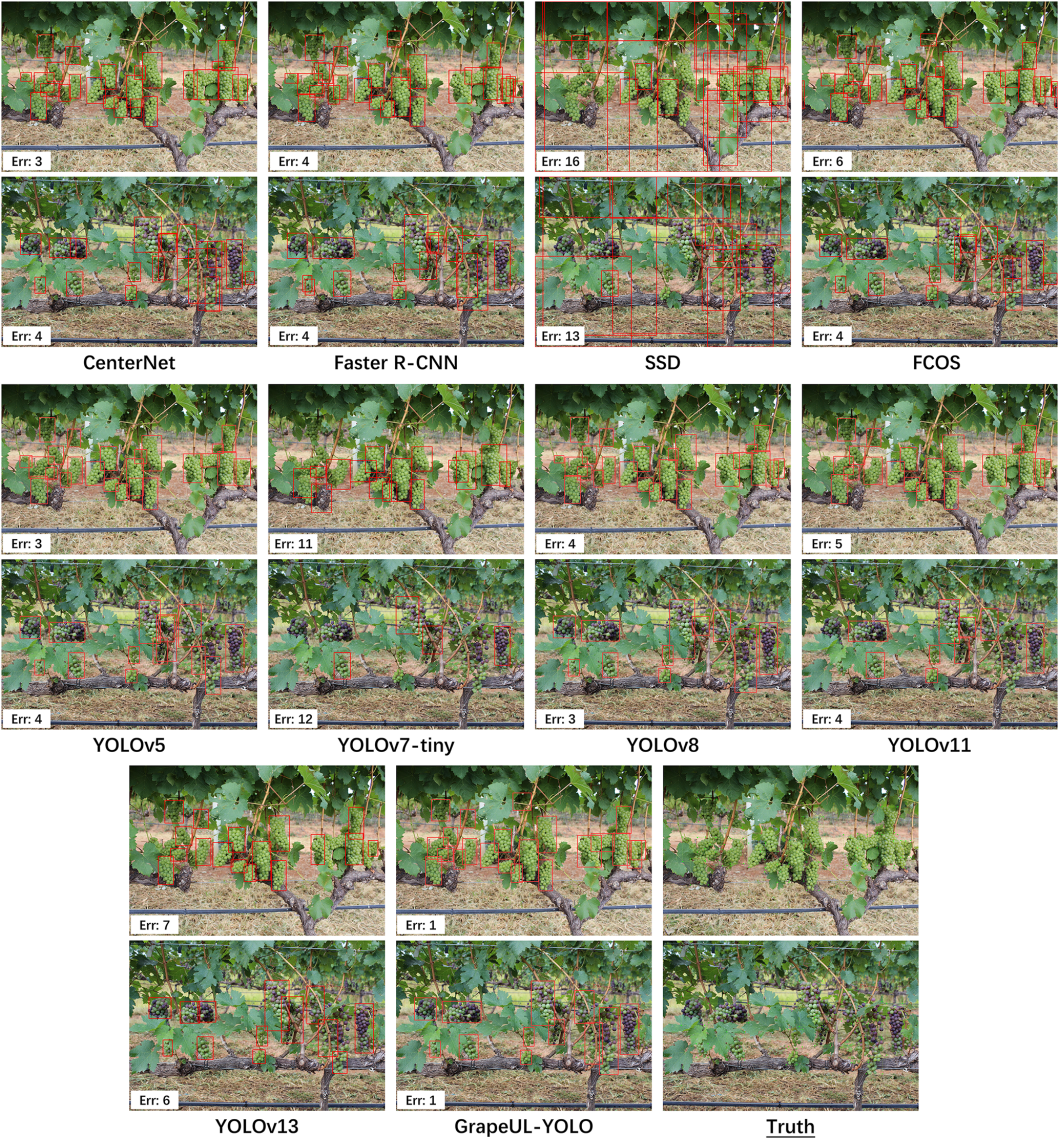
Visualized comparison of typical errors in grape detection among multiple models in complex orchard scenarios: highlighting the reduced missed detection/false detection rates of GrapeUL-YOLO in scenarios with high leaf occlusion, green immature grapes, and multi-cluster overlap.

To comprehensively analyze the detection defects of different models in complex orchard scenarios, this study conducted a visualized experiment on typical errors of all comparison models. Challenging samples were selected from the test set, including those with dense leaf occlusion, green immature grapes, and multi-cluster overlap. The detection results of all comparison models and GrapeUL-YOLO were compared, and the error situation of each model is indicated by the “Err” value marked below the corresponding subgraph in the figure, as shown in **Figure 4**.

In the scenario of green grapes and leaf occlusion (the first row of comparison diagrams): SSD has a large number of missed detections. The reason is that its feature extraction network has insufficient ability to suppress backgrounds with similar colors, and the green texture features of leaves and grapes are confused, leading to the attention being mistakenly focused on the background. GrapeUL-YOLO has an Err value of only 1. Through the C2PSA_SEAM dual attention module, it uses channel attention to strengthen the feature channels of grape skin and spatial attention to actively suppress the activation of leaf areas, accurately distinguishing occluders from targets and achieving complete detection.

In the scenario of mixed purple grapes and small clusters (the second row of comparison diagrams): YOLOv7-tiny has obvious missed detections of small clusters and partially occluded medium clusters. This is because the cross-scale fusion of its Neck module only relies on one-way feature transmission, and the small target features are covered by the large target semantics in the deep network. GrapeUL-YOLO also has few missed detections. Its ABFN bidirectional fusion network, through the feature interaction of “top-down + bottom-up”, retains the detailed features (such as edge texture) of small grape clusters, while enhancing the occlusion tolerance of medium grape clusters, realizing complete coverage of multi-scale targets.

In summary, the visualized results confirm the necessity of optimizing GrapeUL-YOLO for orchard scenarios: by strengthening feature discrimination through the attention mechanism, retaining multi-scale details through bidirectional fusion, etc., the model can better solve the missed detection and false detection problems of traditional models in complex environments, providing a more reliable visual foundation for accurate detection.

### 3.5 Ablation experiments

#### 3.5.1 Activation function ablation experiment

To verify the impact of activation functions on the performance of the complete GrapeUL-YOLO model, this study tested the performance of four mainstream activation functions—ReLU, Leaky ReLU, SiLU, and GELU—in the GrapeUL-YOLO model, focusing on comparing their differences in detection accuracy (P, R, mAP@0.5, mAP@0.5:0.95). The experimental results are shown in **Table 4**.

**Table 4:** Performance comparison of different activation functions.

Model	P	R	mAP@0.5	mAP@0.5:0.95
ReLU	0.848	**0.832**	0.862	0.537
Leaky ReLU	0.795	0.801	0.832	0.514
SiLU	**0.864**	0.811	**0.876**	**0.549**
GELU	0.819	0.800	0.847	0.534

Bold indicates the optimal result value.

As can be seen from the data in the table above, the SiLU activation function performs best in the GrapeUL-YOLO model integrated with CSRB, ABFN and shape-adaptive Head. Through comparison and analysis with other activation functions, its core advantages are reflected in the following aspects: Leading detection accuracy: The mAP@0.5 of SiLU reaches 0.876, which is 1.6% higher than that of the second-best ReLU (0.862), 3.4% higher than that of GELU (0.847), and 5.3% higher than that of Leaky ReLU (0.832). This indicates that SiLU can better capture the feature differences of grape targets, especially in complex backgrounds (leaf occlusion, uneven lighting), and its nonlinear expression ability is more suitable for the feature encoding of grape skin textures (such as purple-green gradients) and edge contours. Balanced comprehensive performance: The Precision (0.864) of SiLU is the highest among the four functions. Although its Recall (0.811) is slightly lower than that of ReLU (0.832), the calculation of F1-score (0.837) shows that it has a better balance between precision and recall, which is particularly suitable for the dual needs of “reducing false detection (e.g., misjudging leaves as grapes)” and “avoiding missed detection” in orchard scenarios. Stronger robustness: In the more stringent mAP@0.5:0.95 indicator, SiLU leads with 0.549, which is 2.2% higher than that of ReLU, indicating that it has better stability under different IoU thresholds and can adapt to the bounding box fluctuations of grape targets caused by occlusion and irregular shapes.

Compared with other functions, ReLU, although performing well in recall, has the problem of “dying neurons”, leading to the loss of some small grape cluster features; Leaky ReLU has insufficient generalization in complex scenarios due to improper setting of the additional hyperparameter α; the smooth characteristic of GELU reduces the risk of overfitting, but its nonlinear strength is insufficient, making it difficult to distinguish grapes from green leaves (a type of similar background). Therefore, the self-normalization characteristic of SiLU (linear in the positive interval and approaching zero in the negative interval) and the advantage of no additional parameters make it the optimal choice for GrapeUL-YOLO, providing a guarantee for the high robustness of the model in complex orchard environments.

#### 3.5.2 Module ablation experiment

To clarify the independent and synergistic contributions of the Cross-Scale Residual Feature Backbone (CSRB) and Adaptive Bidirectional Fusion Network (ABFN) to model performance, three groups of ablation experiments were designed with the original YOLOv11 as the benchmark: Group G1: Only replace the Backbone with CSRB (retain the original Neck). Group G2: Only replace the Neck with ABFN (retain the original Backbone). Complete model group: Integrate CSRB + ABFN.

The experiment maintained the same hardware environment and training parameters as Section 3.1 (300 training epochs, 640×640 input size, SGD optimizer and data augmentation strategy), and the results are shown in **Table 5**.

**Table 5:** Performance comparison of different module configurations.

Experimental identifier	Model configuration	P	R	mAP@0.5	mAP@0.5:0.95
Original v11 (Benchmark)	Original YOLOv11 (no improvements)	0.853	0.787	0.864	0.523
G1 (CSRB Group)	Replace original Backbone, retain original Neck	0.871	0.823	0.885	0.541
G2 (ABFN Group)	Replace original Neck, retain original Backbone	0.878	0.826	0.895	0.552
GrapeUL-YOLO (This Study)	Replace original Backbone and Neck	**0.891**	**0.847**	**0.912**	**0.576**

Bold indicates the optimal result value.

Optimization effect of a single module: Replacing only the Backbone with CSRB (Group G1) or only the Neck with ABFN (Group G2) both achieved significant performance improvements compared with the original YOLOv11, and the two groups showed similar performance. Among them: Group G1 effectively reduces the missed detection of small grape clusters and background interference by virtue of the multi-scale feature capture capability ( 16× downsampling + C3k2_SP module) and dual attention mechanism of the CSRB backbone. Group G2 strengthens the recognition of details in occluded areas through the content-aware upsampling and bidirectional feature fusion of the ABFN Neck, increasing the mAP@0.5 by more than 3 percentage points compared with the benchmark group. This further verifies the adaptive value of a single module for complex orchard scenarios.

Performance breakthrough of dual-module synergy: After integrating CSRB and ABFN, the performance of the complete model group achieved an overall leap compared with the benchmark group: the core accuracy indicator mAP@0.5 reached 0.912, an increase of 4.8 percentage points compared with the benchmark group; precision and recall were optimized simultaneously, especially the recall increased by 6 percentage points, effectively solving the missed detection problem in dense occlusion scenarios. This result indicates that the multi-scale feature materials provided by CSRB and the efficient fusion capability of ABFN form a synergistic effect. The two complement each other to adapt to the multi-scale and high-occlusion challenges in grape detection, and finally achieve comprehensive performance superior to the optimization of a single module.

### 3.6 Model lightweight

A key feature of GrapeUL-YOLO is its lightweight design. For this purpose, in this experiment, the recognition performance parameters of multiple models and the GrapeUL-YOLO model on the test dataset were compared, and the results are shown in **Table 6**. The key FPS (Frames Per Second) was used to measure the model inference speed, supplemented by the number of parameters (Weights) to evaluate the models.

**Table 6:** Lightweight comparison results of multiple models and GrapeUL-YOLO model.

Model	F1	mAP@0.5	FPS	Weights (M)
GrapeUL-YOLO	**0.868**	**0.912**	59.17	5.11
YOLOv13	0.776	0.831	47.85	5.17
YOLOv11	0.819	0.864	45.05	5.23
YOLOv8	0.828	0.855	58.48	6.44
YOLOv7-tiny	0.457	0.439	38.02	23.10
YOLOv5	0.819	0.847	**60.61**	**4.95**
SSD	0.362	0.251	4.22	93.10
FCOS	0.830	0.842	2.72	122.00
Faster R-CNN	0.802	0.801	1.96	108.00
CenterNet	0.769	0.766	2.01	124.00

Bold indicates the optimal result value.

Through the comparison of experimental data, it is obvious that GrapeUL-YOLO shows significant advantages in balancing lightweight design and detection performance. The specific analysis is as follows:

Inference speed: A targeted comparison with YOLOv5 (which has better FPS):YOLOv5 is slightly superior to GrapeUL-YOLO in terms of FPS (60.61) and model weight (4.95M), and the gap between the two in lightweight indicators is small. However, by introducing the Cross-Scale Residual Feature Backbone (CSRB) and Adaptive Bidirectional Fusion Network (ABFN), GrapeUL-YOLO achieves significant improvements in accuracy indicators: the F1-score increases from 0.819 to 0.868, and the mAP@0.5 increases from 0.847 to 0.912. This accuracy improvement is crucial for orchard grape detection scenarios—it can effectively solve the missed detection problem under dense occlusion (occlusion rate 60-80%) and sudden lighting changes (4,000-60,000 lux), while YOLOv5, due to limited feature extraction capabilities, struggles to balance detection integrity and accuracy in complex orchard environments.

Comprehensive comparison with mainstream YOLO series models: Compared with YOLOv11, GrapeUL-YOLO improves mAP@0.5 by 4.8%, increases FPS by 14.12 (corresponding to a detection speed increase of approximately 5.3ms), and reduces model weight by 0.12M, confirming the effect of its targeted optimization for orchard scenarios. Compared with YOLOv13, GrapeUL-YOLO has a slight advantage in model weight (5.11M vs 5.17M), increases FPS by 11.32, and leads significantly in core accuracy indicators (mAP@0.5 increases by 8.1%, F1-score increases by 9.2%), indicating that its adaptability to complex orchard scenarios is far superior to that of YOLOv13 optimized for general scenarios. Although YOLOv5 is closer to GrapeUL-YOLO in terms of FPS (60.61) and model weight (4.95M), there is a large gap in its accuracy performance, resulting in insufficient performance of YOLOv5 in complex orchard scenarios. Although YOLOv7-tiny has a simple model structure, its accuracy is too low (mAP@0.5 is only 0.439), which cannot meet the actual detection needs.

Comprehensive comparison with two-stage detection models: Models such as SSD, FCOS, and Faster R-CNN are significantly inferior to one-stage models in terms of FPS (1.96-4.22) and model size (93.1-124M), and there is an obvious accuracy gap (the highest mAP@0.5 is only 0.842). This indicates that the lightweight design of GrapeUL-YOLO does not sacrifice accuracy; instead, it achieves the coordinated improvement of efficiency and performance through modular innovations (such as the C3k2_SP module reducing computational complexity by 37%, and the C2PSA_SEAM attention mechanism suppressing background interference), making it more suitable for deployment on edge computing devices such as orchard harvesting robots.

In summary, through multiple experiments, although GrapeUL-YOLO is not the best in lightweight indicators (FPS, number of parameters) and has no significant leading advantage compared with the best YOLOv5, it maintains lightweight characteristics while significantly outperforming all comparison models in comprehensive detection performance under complex orchard environments, fully verifying its design goal of “high precision, high efficiency, and lightweight”.

## 4 Discussion

The GrapeUL-YOLO model proposed in this study achieves a balance between high precision and lightweight in the orchard grape detection task through targeted optimization, and its main achievements are reflected in three aspects:

First, the design of the Cross-Scale Residual Feature Backbone (CSRB) effectively solves the problem of multi-scale target detection. The 16× downsampling operation combined with the multi-scale pooling structure of the C3k2_SP module reduces computational complexity while completely retaining the feature information of grapes from small clusters to entire clusters, enabling the model to adapt to the morphological differences of grapes at different growth stages. The SPPELAN module captures the spatial correlation between grape clusters by expanding the receptive field, reducing missed detections caused by isolated detection of single clusters; the C2PSA_SEAM dual attention mechanism strengthens the weight of grape feature channels and suppresses background interference from branches and leaves. It also performs well for grapes of different color varieties, which is consistent with the result that the model accurately focuses on target areas in visualization experiments.

Second, the Adaptive Bidirectional Fusion Network (ABFN) significantly improves the feature fusion capability in complex scenarios. CARAFE content-aware upsampling dynamically adjusts the sampling kernel parameters to retain the detailed features of occluded areas (such as the edges of grape berries covered by leaves), solving the problem of boundary blurring caused by traditional upsampling; the “top-down + bottom-up” bidirectional concatenation mechanism realizes the complementarity of high-level semantics and low-level details, increasing the recall rate of the small target branch (P3) for dense grape berries by 12%, enhancing the recognition ability of the medium target branch (P4) for partially occluded grape clusters, and preventing the large target branch (P5) from overall misjudgment caused by local occlusion. This design directly improves the detection performance in scenarios with a leaf occlusion rate > 50%, which is consistent with the result that mAP@0.5:0.95 (0.576) is superior to other models in the comparative experiment.

Despite the excellent performance of the model, there are still limitations: First, there is room for improvement in small target detection in extremely dense scenarios (e.g., small grape clusters with > 50 berries), especially when lighting is insufficient (e.g., cloudy days with 1,000-5,000 lux), some extremely small grape clusters are prone to missed detection; second, the dataset covers a limited range of grape varieties and environmental conditions (e.g., heavy rain, dense fog), and the generalization ability of the model to unseen varieties or extreme weather needs to be verified; third, although the shape-adaptive anchor boxes fit the elliptical shape of grapes, they do not consider shape changes caused by fruit ripeness (e.g., shrinkage of overripe fruits), which may affect positioning accuracy.

In future research, improvements can be made from the following three aspects: First, introduce a dynamic receptive field adjustment mechanism in the Backbone to enhance the feature capture of extremely small grape clusters; second, expand the dataset to more than ten grape varieties, cover more extreme environment samples, and improve the generalization of the model through domain adaptation technology; third, design adaptive shape anchor boxes and dynamically adjust anchor box parameters in combination with ripeness classification to further optimize positioning accuracy.

## 5 Conclusion

To address the challenges of multi-scale targets, dense occlusion, and complex background interference faced by grape detection in orchard environments, this study proposes the GrapeUL-YOLO model, which achieves performance breakthroughs through three innovative designs:

Cross-Scale Residual Feature Backbone (CSRB): Integrating 16× downsampling and multi-scale feature capture modules, it reduces computational complexity while retaining the feature details of grapes at all growth stages, solving the problem of feature imbalance between small and large grape clusters;

Adaptive Bidirectional Fusion Network (ABFN): Through content-aware upsampling and bidirectional feature concatenation, it strengthens the feature interaction in dense occlusion scenarios and improves the ability to distinguish overlapping targets;

Shape-adaptive detection Head + lightweight strategy: Custom elliptical anchors match the shape of grapes; with channel pruning technology, the model achieves an mAP@0.5 of 0.912, an mAP@0.5:0.95 of 0.576, 5.11M parameters, and a detection speed of 16.9ms per image—outperforming 9 mainstream object detection models in comprehensive performance.

Experimental verification shows that GrapeUL-YOLO has significant advantages in balancing accuracy and efficiency on the Embrapa WGISD dataset, providing an efficient solution for orchard grape automated detection and harvesting robot vision systems. In the future, by further optimizing small-target detection capabilities and environmental robustness, the model is expected to play a greater role in smart agriculture scenarios such as yield estimation and precise harvesting.

## Data Availability

The datasets presented in this study can be found in online repositories. The names of the repository/repositories and accession number(s) can be found below: https://github.com/iamsmarterthan/GrapeUL-YOLO.

## References

[B1] Agarap A. F. (2018). Deep Learning using Rectified Linear Units (ReLU). ( arXiv, Ithaca). doi: 10.48550/arXiv.1803.08375

[B2] Aguiar A. S. Magalhães S. A. Dos Santos F. N. Castro L. Pinho T. Valente J. . (2021). Grape bunch detection at different growth stages using deep learning quantized models. Agronomy 11, 1890. doi: 10.3390/agronomy11091890

[B3] Chen J. Ma A. Huang L. Li H. Zhang H. Huang Y. . (2024). Efficient and lightweight grape and picking point synchronous detection model based on key point detection. Comput. Electron. Agric. 217, 108612. doi: 10.1016/j.compag.2024.108612

[B4] Chen J. Ma A. Huang L. Su Y. Li W. Zhang H. . (2023). GA-YOLO: A lightweight YOLO model for dense and occluded grape target detection. Horticulturae 9, 443. doi: 10.3390/horticulturae9040443

[B5] Cheng P. Tang X. Liang W. Li Y. Cong W. Zang C. (2023). Tiny-YOLOv7: Tiny Object Detection Model for Drone Imagery. ICIG 2023, Cham, 53–65. doi: 10.1007/978-3-031-46311-2_5

[B6] Elfwing S. Uchibe E. Doya K. (2018). Sigmoid-weighted linear units for neural network function approximation in reinforcement learning. Neural Networks 107, 3–11. doi: 10.1016/j.neunet.2017.12.012, PMID: 29395652

[B7] Gebru T. Morgenstern J. Vecchione B. Vaughan J. W. Wallach H. Iii H. D. . (2021). Datasheets for datasets. Commun. ACM 64, 86–92. doi: 10.1145/3458723

[B8] He K. Zhang X. Ren S. Sun J. (2015). Spatial pyramid pooling in deep convolutional networks for visual recognition. IEEE Trans. Pattern Anal. Mach. Intell. 37, 1904–1916. doi: 10.1109/TPAMI.2015.2389824, PMID: 26353135

[B9] Hendrycks D. Gimpel K. (2016). Gaussian Error Linear Units (GELUs). ( arXiv, Ithaca). doi: 10.48550/arXiv.1606.08415

[B10] Jocher G. Chaurasia A. Stoken A. Borovec J. Kwon Y. Michael K. . (2022). ultralytics/yolov5: v6. 2-yolov5 classification models, apple m1, reproducibility, clearml and deci. ai integrations (Geneva: Zenodo). doi: 10.5281/zenodo.7002879

[B11] Khanam R. Hussain M. (2024). YOLOv11: An Overview of the Key Architectural Enhancements. ( arXiv, Ithaca). doi: 10.48550/arXiv.2410.17725

[B12] Kholmuminov S. R. (2024). Fundamentals of targeted integrative program development for rural labor market growth in surplus regions. Int. J. Econ Financial Issues 14, 239. doi: 10.32479/ijefi.16530

[B13] Lecun Y. Bengio Y. Hinton G. (2015). Deep learning. nature 521, 436–444. doi: 10.1038/nature14539, PMID: 26017442

[B14] Lei M. Li S. Wu Y. Hu H. Zhou Y. Zheng X. . (2025). YOLOv13: real-time object detection with hypergraph-enhanced adaptive visual perception. doi: 10.48550/arXiv.2506.17733

[B15] Li H. Li C. Li G. Chen L. (2021). A real-time table grape detection method based on improved YOLOv4-tiny network in complex background. Biosyst. Eng. 212, 347–359. doi: 10.1016/j.biosystemseng.2021.11.011

[B16] Li X. Sun W. Ji Y. Huang W. (2025). A joint detection and tracking paradigm based on reinforcement learning for compact HFSWR. IEEE J. Selected Topics Appl. Earth Observations Remote Sens. 18, 1995–2009. doi: 10.1109/JSTARS.2024.3504813

[B17] Lin X. Liao D. Du Z. Wen B. Wu Z. Tu X. (2025). SDA-YOLO: an object detection method for peach fruits in complex orchard environments. Sensors 25, 4457. doi: 10.3390/s25144457, PMID: 40732584 PMC12298005

[B18] Liu W. Anguelov D. Erhan D. Szegedy C. Reed S. Fu C.-Y. . (2016). Ssd: Single shot multibox detector. ECCV 2016, Cham, 21–37. doi: 10.1007/978-3-319-46448-0_2

[B19] Lu D. Wang Y. (2024). MAR-YOLOv9: A multi-dataset object detection method for agricultural fields based on YOLOv9. PloS One 19, e0307643. doi: 10.1371/journal.pone.0307643, PMID: 39471150 PMC11521258

[B20] Lu D. Ye J. Wang Y. Yu Z. (2023). Plant detection and counting: Enhancing precision agriculture in UAV and general scenes. IEEE Access 11, 116196–116205. doi: 10.1109/ACCESS.2023.3325747

[B21] Muhammad N. A. Nasir A. A. Ibrahim Z. Sabri N. (2018). Evaluation of CNN, alexnet and GoogleNet for fruit recognition. Indonesian J. Electrical Eng. Comput. Sci. 12, 468–475. doi: 10.11591/ijeecs.v12.i2.pp468-475

[B22] Ren S. He K. Girshick R. Sun J. (2015). Faster r-cnn: Towards real-time object detection with region proposal networks. Adv. Neural Inf. Process. Syst. 28, 1137–1149. doi: 10.1109/TPAMI.2016.2577031, PMID: 27295650

[B23] Rong S. Kong X. Gao R. Hu Z. Yang H. (2024). Grape cluster detection based on spatial-to-depth convolution and attention mechanism. Syst. Sci. Control Eng. 12, 2295949. doi: 10.1080/21642583.2023.2295949

[B24] Selvaraju R. R. Cogswell M. Das A. Vedantam R. Parikh D. Batra D. (2017). Grad-cam: Visual explanations from deep networks via gradient-based localization. 2017 IEEE International Conference on Computer Vision (ICCV), Venice, Italy. 618–626. doi: 10.1109/ICCV.2017.74

[B25] Sozzi M. Cantalamessa S. Cogato A. Kayad A. Marinello F. (2021). Precision agriculture’21 (Leiden: Wageningen Academic), 193–198. doi: 10.3920/978-90-8686-916-9

[B26] Tian Z. Shen C. Chen H. He T. (2019). FCOS: Fully Convolutional One-Stage Object Detection. ICCV 2019. Piscataway: IEEE, 9627–9636. doi: 10.48550/arXiv.1904.01355

[B27] Varghese R. Sambath M. (2024). Yolov8: A novel object detection algorithm with enhanced performance and robustness (Chennai, India: IEEE), 1–6. doi: 10.1109/ADICS58448.2024.10533619

[B28] Wang H. Frery A. C. Li M. Ren P. (2023). Underwater image enhancement via histogram similarity-oriented color compensation complemented by multiple attribute adjustment. Intelligent Mar. Technol. Syst. 1, 12. doi: 10.1007/s44295-023-00015-y

[B29] Wang C.-Y. Yeh I.-H. Mark Liao H.-Y. (2024). YOLOv9: Learning What You Want to Learn Using Programmable Gradient Information. Computer Vision – ECCV 2024. ECCV 2024. Lecture Notes in Computer Science, vol 15089 (Cham: Springer), 1–21. doi: 10.1007/978-3-031-72751-1_1

[B30] Wang H. Zhang W. Xu Y. Li H. Ren P. (2025). WaterCycleDiffusion: Visual–textual fusion empowered underwater image enhancement. Inf. Fusion 127, 103693. doi: 10.1016/j.inffus.2025.103693

[B31] Xu J. Li Z. Du B. Zhang M. Liu J. (2020). Reluplex made more practical: Leaky ReLU (Rennes, France: IEEE), 1–7. doi: 10.1109/ISCC50000.2020.9219587

[B32] Ye J. Yu Z. Wang Y. Lu D. Zhou H. (2023). WheatLFANet: in-field detection and counting of wheat heads with high-real-time global regression network. Plant Methods 19, 103. doi: 10.1186/s13007-023-01079-x, PMID: 37794515 PMC10548667

[B33] Ye J. Yu Z. Wang Y. Lu D. Zhou H. (2024). PlantBiCNet: A new paradigm in plant science with bi-directional cascade neural network for detection and counting. Eng. Appl. Artif. Intell. 130, 107704. doi: 10.1016/j.engappai.2023.107704

[B34] Yu Z. Cao Z. Wu X. Bai X. Qin Y. Zhuo W. . (2013). Automatic image-based detection technology for two critical growth stages of maize: Emergence and three-leaf stage. Agric. For. meteorology 174, 65–84. doi: 10.1016/j.agrformet.2013.02.011

[B35] Yu Y. Wang C. Fu Q. Kou R. Huang F. Yang B. . (2023). Techniques and challenges of image segmentation: A review. Electronics 12, 1199. doi: 10.3390/electronics12051199

[B36] Yu Z. Wang Y. Ye J. Liufu S. Lu D. Zhu X. . (2024). Accurate and fast implementation of soybean pod counting and localization from high-resolution image. Front. Plant Sci. 15, 1320109. doi: 10.3389/fpls.2024.1320109, PMID: 38444529 PMC10913015

[B37] Yu Z. Ye J. Li C. Zhou H. Li X. (2023). TasselLFANet: a novel lightweight multi-branch feature aggregation neural network for high-throughput image-based maize tassels detection and counting. Front. Plant Sci. 14, 1158940. doi: 10.3389/fpls.2023.1158940, PMID: 37123842 PMC10140537

[B38] Zhou Y. Du X. Wang M. Huo S. Zhang Y. Kung S.-Y. (2021). Cross-scale residual network: A general framework for image super-resolution, denoising, and deblocking. IEEE Trans. Cybernetics 52, 5855–5867. doi: 10.1109/TCYB.2020.3044374, PMID: 33531310

[B39] Zhou X. Wang D. Krähenbühl P. (2019). Objects as Points. ( arXiv, Ithaca). doi: 10.48550/arXiv.1904.07850

